# Partnering with postdocs: a library model for supporting postdoctoral researchers and educating the academic research community

**DOI:** 10.5195/jmla.2020.902

**Published:** 2020-07-01

**Authors:** Karen H. Gau, Pamela Dillon, Teraya Donaldson, Stacey Elizabeth Wahl, Carrie L. Iwema

**Affiliations:** 1 gaukh@vcu.edu, Health Sciences Collection Librarian, Tompkins-McCaw Library for the Health Sciences, Virginia Commonwealth University, Richmond, VA; 2 pmdillon@vcu.edu, Research Liaison, C. Kenneth and Dianne Wright Center for Clinical and Translational Research, Virginia Commonwealth University, Richmond, VA; 3 donaldson.teraya@nih.gov, Assistant Director of Education, C. Kenneth and Dianne Wright Center for Clinical and Translational Research, Virginia Commonwealth University, Richmond, VA; 4 swahl@vcu.edu, Research and Education Librarian, Tompkins-McCaw Library for the Health Sciences, Virginia Commonwealth University, Richmond, VA; 5 iwema@pitt.edu, Coordinator of Basic Science Services, Health Sciences Library System, University of Pittsburgh, Pittsburgh, PA

## Abstract

**Background:**

A mutually beneficial need exists between postdoctoral scholars (postdocs) who want to grow their science communication, networking, and teaching skills and those in the general health sciences research community who want to learn more about specialized topics. Recognizing this need, interdepartmental teams at two public universities began offering postdocs a teaching opportunity at their health sciences libraries, which serve as discipline-neutral learning spaces for researchers.

**Case Presentation:**

At the University of Pittsburgh (Pitt) and Virginia Commonwealth University (VCU), postdocs are invited to submit talk proposals on “how to do something” related to the health sciences. Selected postdoc speakers conduct one-hour talks, get science communication and teaching support, have their talks uploaded to YouTube, and receive feedback from attendees.

**Conclusions:**

Postdoc participants appreciated being able to participate in this program, and attendees strongly indicated that the talks are of value. At VCU, surveys of the 25 talks from 2015–2018 showed that 91% of attendees believed they had a better understanding of the topic because of their attendance, and 85% planned to use the knowledge they gained. More than a year after their talks, several postdocs across both institutions informed the coordinators that they were subsequently contacted for advice or further discussion, with 2 postdocs stating that it helped them with job opportunities. This model can be easily adapted at other health sciences libraries to benefit their academic communities.

## BACKGROUND

A postdoctoral scholar (postdoc) is an individual “who has received a doctoral degree (or equivalent) and is engaged in a temporary and defined period of mentored advanced training to enhance the professional skills and research independence needed to pursue his or her chosen career path” [1]. A 2010 and 2013 survey of biological and life sciences postdocs indicated that 60% of respondents pursued postdoc positions with the goal of securing tenure-track faculty positions [2], but in general, fewer than a third of all postdocs were able to do so [3–5]. Thus, it is important for postdocs to be prepared for a broad range of careers. Postdocs can meet this challenge by using their time as advanced trainees to expand their skill sets beyond those required for their immediate research, such as developing their science communication, networking, and teaching skills, which can be useful in both academia and industry [6–10]. The National Postdoctoral Association also recognizes “Communication Skills” and “Professionalism” as two of their six core competencies that are “critical for development during postdoctoral appointments” [11].

Health sciences libraries are uniquely positioned to support postdocs in these areas. Libraries are often able to provide teaching spaces that their academic communities recognize as discipline neutral [12–14], which situates them as ideal locations for teaching interdisciplinary topics. Providing postdocs with an interdisciplinary teaching opportunity affords them an opportunity to develop the aforementioned skill sets and expand their networking reach. Additionally, many liaison librarians are experienced in communicating, networking, and teaching [15, 16] and can offer focused assistance to postdocs in these areas, allowing them to reach out to a largely untapped user group [17]. Partnering with other university resources, such as postdoctoral associations or translational research centers, can enhance networking and science communication support for postdocs, while also strengthening institutional collaborations.

Moreover, providing postdocs with an interdisciplinary space to share their specialized knowledge with the academic community falls in line with academic libraries' overall goals of advancing knowledge and research at their institutions. For example, the University of Pittsburgh (Pitt) Health Sciences Library System “advance[s] learning, teaching, research, and service across the health sciences community” [18], and Virginia Commonwealth University (VCU) Libraries' 2020 strategic framework states that one of its goals is to “improve health and well-being,” in part by “[f]acilitat[ing] and support[ing] connections to enhance interdisciplinary research on health and well being” [19].

Health sciences librarians at Pitt and VCU and translational research faculty at VCU sought to provide a professional development platform for postdocs to teach and practice their general communication and networking skills, which would also serve to advance health sciences research in their academic communities. To that end, Pitt and VCU have been offering a “How-to Talks by Postdocs” series at their respective institutions, where postdocs are invited to conduct instructional talks on health sciences topics of their choice. The talks are focused on how to do something related to the health sciences and are not seminar talks on specific research projects. Postdoctoral associations at both institutions have been supporting partners of the series.

Although programs dedicated to postdoc professional development exist [20–22], this series is a low-commitment alternative for postdocs that can increase their exposure and enhance their curriculum vitae (CVs). The time commitment for attendees is minimal and potentially beneficial.

## CASE PRESENTATION

Pitt launched the “How-to Talks by Postdocs” series in 2013 and has been hosting a session annually since then, with a total of 32 talks and 242 attendees by the end of 2018. VCU launched its fall series in 2015 and hosted 25 talks with 476 attendees through 2018. Attendance at the talks is generally capped at 25 or 45, depending on the room and/or topic, so that the postdocs have a manageable teaching environment. Hands-on programming trainings, for instance, might be limited to fewer attendees. Covered topics have concerned specific techniques, analyses, professional development, and programming (Table 1).

**Table 1:** Selection of talks included in the “How-to Talks by Postdocs” series

Topic	Talks
Techniques/protocols	“Using 3D Printing for Tissue Engineering” “CRISPR/Cas9 Genome Editing” “How to Grow Cancer Cells as Free-Floating Tumor Spheroids” “Manipulation of Promiscuous Micrornas in the Cell”
Analysis	“How to Analyze Protein Expression in Cell Culture and Tissue Specimens” “Causal Analysis: How to Examine Mediation and Moderation of Treatment or Experimental Effects” “How to Conduct a Biomechanical Analysis of Human Movement”
Professional development	“Managing Multiple Collaborations” “The Active Learning Teaching Revolution: Teaching Science, Technology, Engineering, Math (STEM) so That Your Students Actually Learn” “How to Prepare a CV (Grad Students and Postdocs)” “How to Obtain a Postdoc”
Programming	“UNIX for Biologists” “Intro to R-Package” “Introduction to RNA-Seq Analysis with an Example in R” “Curve Fitting in MATLAB”

### Who are partners to the series?

At both institutions, the series is coordinated by the health sciences libraries. The Wright Center for Clinical and Translational Research also shares coordinating responsibilities at VCU. Series coordination involves marketing to postdocs and potential attendees; reviewing postdoc speaker applications; providing feedback for postdoc practice sessions; and coordinating logistics, such as the series schedule and room reservations. The postdoctoral associations at both institutions are marketing partners. All talks in the series are hosted at the health sciences libraries because they serve as discipline-neutral spaces. These partnerships are critical for spreading the workload (VCU), increasing the series' marketing reach, and providing broad support for postdocs at each university.

### How does it work?

The VCU series was originally modeled after the one at Pitt. Before initiating their program, the VCU coordinators conducted a focus group with several postdocs to identify the university's unique needs prior to launching the series. The focus group participants answered questions about the scheduling, marketing, and type of support postdocs would want for their presentations. In return for their participation, the postdocs received a free lunch. Decisions about how to proceed with VCU's inaugural program were based on focus group feedback.

At Pitt and VCU, postdocs are asked to submit proposals for one-hour talks about health sciences topics a few months prior to the start of each series. Coordinators' suggestions may be included to try to recruit for desired topics. Multiple proposals may be submitted by a single postdoc. Pitt has an informal application process: interested postdocs email the coordinator and initiate a dialogue about presentation topics and scheduling. VCU requires submission of an online application that includes a two-to-three sentence description of the proposed talk and the postdoc's experience on the subject matter. The coordinators then determine which talks will be included as part of that semester's series, which generally includes four to seven talks. Predetermined criteria for selection include the likelihood that a topic will attract an audience—topics that appear to be trending in the scientific community or at the institutions are prioritized—as well as the expectation that a talk will broaden the scope of the series. As an example of the latter, if there are more applications than available lecture slots and many of the applications are related to molecular biology, then a talk proposal related to engineering may be more strongly considered.

Depending on the institution, postdocs work with the program coordinator to schedule talks during mutually agreeable times (Pitt) or are assigned to consistent, fixed time slots to aid with advertising the series (VCU). As part of the program, postdoc speakers are provided (a) letters of acknowledgment for their participation, (b) the opportunity to conduct practice runs and obtain feedback prior to their scheduled presentations, (c) optional video-recordings of their presentations for self-assessment and/or upload to YouTube, and (d) feedback on their presentations from both the coordinators and attendees. A teaching workshop may be offered prior to the start of the series to help support postdocs. For instance, during the launch of the fall series at VCU, postdoc speakers are invited to attend a workshop on best teaching practices. Attendance at Pitt's program is first-come, first-served, while VCU requests prior registration.

### How is the series marketed?

The “How-to Talks by Postdocs” series is advertised through a variety of media, including physical and digital signage, blog articles, newsletters, university and departmental event calendars, email list distribution, and table tents. Pitt has a website for its talks [23], as does VCU [24]. VCU's attendee surveys of the 25 fall talks that it hosted from 2015–2018 (supplemental Appendix A) indicate that the most successful marketing method is through email distribution to postdocs, graduate students, staff, and faculty from the health sciences schools, centers, and institutes as well as the biology, chemistry, and engineering schools (59%, 235 of 396 respondents). VCU also markets its series to the biomedical research community outside of the university.

### Who attends? Are the talks useful to them?

**Figure 1:**
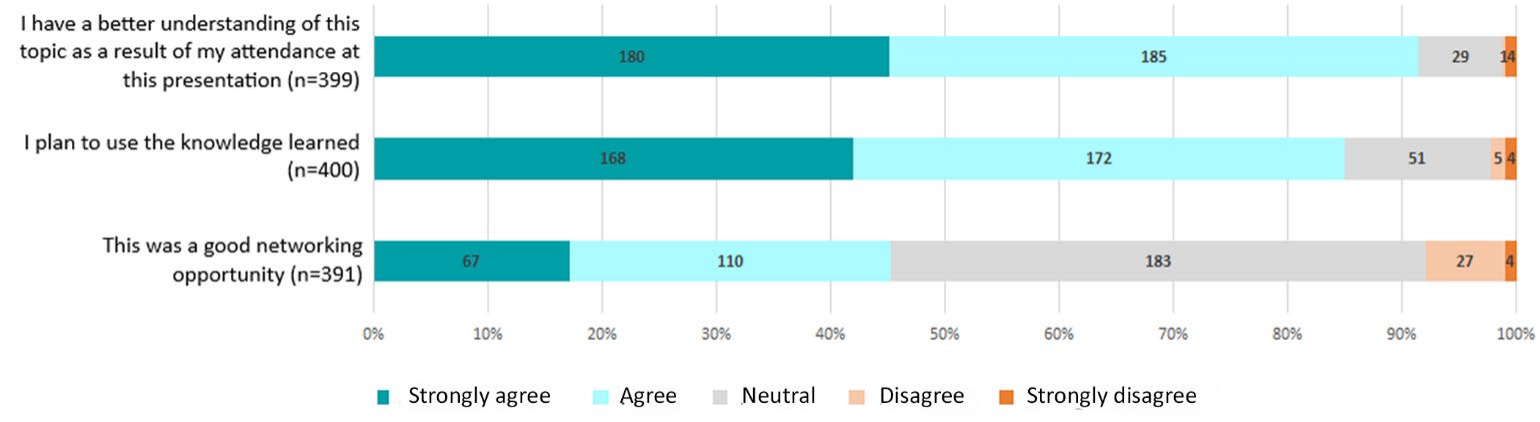
Aggregated attendee feedback from Virginia Commonwealth University's (VCU's) 2015–2018 fall series

Attendees come from varied backgrounds and have included faculty, staff, postdocs, and students from basic science, various clinical disciplines, allied health fields, mathematics, and engineering departments, centers, and institutes. VCU also experimented with offering Hampton University limited remote access to its talks in fall 2017, although this was discontinued due to lack of participation. VCU's 2015–2018 attendee surveys indicate that 91% of respondents (365 of 399) agreed or strongly agreed that they had a better understanding of the topic because of their attendance, and 85% (340 of 400) agreed or strongly agreed that they planned to use the knowledge they learned (Figure 1). Nearly half (45%, 177 of 391) agreed or strongly agreed that the series was a good networking opportunity. Due to limited support personnel and the small size of their program, Pitt has not yet performed any formal assessments.

Most talks are recorded and shared on YouTube [25, 26]. The number of views for each talk tend to be low or modest, but there are a few exceptions. One Pitt talk, “How to Identify a Gene of Interest from Exome Sequencing,” has been viewed over 10,500 times on YouTube since December 2014, and a VCU talk, “How to Study Protein-Ligand Interaction through Molecular Docking,” was uploaded in October 2016 and has since generated over 112,000 views. A few other talks have generated more than 1,000 views since they were uploaded.

### What do the postdoc speakers think of the series?

At both institutions, postdoc speakers expressed appreciation for being given a platform to teach, receiving feedback on their presentations, and being able to add this experience to their CVs. All of the postdocs (n=22) who responded to an evaluation of VCU's fall series from 2015–2018 (supplemental Appendix B) indicated that they would participate again and recommend it to other postdocs. About three-quarters (17 of 22) felt that the series was a useful networking opportunity. In fact, the VCU postdocs who presented “Connecting the Dots: Using Backwards Design to Create Cohesive Instruction” were invited afterward by the Center for Teaching and Learning Excellence (CTLE) to give the same talk as part of CTLE's Teaching Excellence Academy. Additionally, at Pitt, a postdoc talk, “Ethical Considerations for Scientific Image Manipulation,” generated interest from the Responsible Conduct of Research (RCR) division of the Clinical and Translational Science Institute and was subsequently included in the RCR's roster of talks.

More than one year after their talks, several postdocs across both institutions informed the coordinators that they were subsequently contacted for advice or further discussion. One postdoc stated that the talk resulted in a new job interview, while another expressed her belief that her participation in the series and ability to direct interviewers to her “How-to Talks by Postdocs” videos were contributing factors to her success in obtaining an industry position that required strong communication skills.

## DISCUSSION

Overall, the collected feedback shows that attendees find the talks useful and that postdoc speakers find value in participating in the series, with some speakers able to leverage it for networking or job opportunities. It is encouraging to note that several postdocs have reported conversations with other researchers following their presentations, suggesting the potential for these talks to stimulate research collaborations and successfully support the National Postdoctoral Association's “Communication Skills” and “Professionalism” core competencies [11]. The coordinators have also increased their exposure to postdocs and other groups in their academic communities through broad marketing of the talks.

Because there are no speaker or space fees involved at either institution, the coordinators consider the financial cost to be relatively low and worthwhile for an ongoing speaker series. The series does, however, require reservation of library spaces, some funding for refreshments (VCU), and a considerable amount of time devoted to coordination and marketing. The amount of staff time that can be dedicated to the series impacts the robustness of the program. Pitt has only one coordinator overseeing the program, whereas VCU has multiple coordinators from different organizations, allowing them to commit more time and support to the series. For instance, VCU has enough staff participation to be able to require practice runs for all its postdoc speakers and to process print surveys filled out by their talk attendees.

### Challenges

Although postdoc speakers have expressed strong enthusiasm for the series, proposals for talks have not been abundant at either institution. Informal feedback regarding the reason for this mainly centers on concerns about taking time to develop a presentation that may have limited or no interest. Some postdocs have suggested to the coordinators that they might be more willing to present for the series if they were given predefined topics for which there was definitive interest. Accordingly, both institutions now suggest topics for talks on their websites. VCU also implemented a science communication award with a financial incentive as part of its 2019 program, the results of which are currently being evaluated. Future considerations for postdoc recruitment include highlighting the program's potential for supporting the National Postdoctoral Association's core competencies [11] and taking a closer look at how the program might help postdocs from underrepresented populations, who face additional challenges [27].

Marketing the series is not a straightforward process. The underlying theme of the series is that postdocs are teaching something related to the health sciences, but the topics vary quite widely. This variety means that the most effective way to market this series is to advertise it broadly during its launch and then strategically advertise each talk thereafter, which is time consuming. Working with postdoc speakers to edit their talk descriptions so that they are understandable to a broader audience can take some time as well.

Another challenge that institutions may face includes finding one or more coordinators with enough science expertise to be able to judge which applications will interest their researchers and to help postdoc speakers find the right balance between explaining a specialized topic and speaking to a broader audience, if a practice run is offered. Both Pitt and VCU's programs include coordinators with science backgrounds.

Lastly, the impact the series has in helping postdocs compete in the job market is unclear. In part, this is because following up with postdoc speakers well after their participation in the series has seen mixed success. Presenters have, however, requested that their YouTube videos be posted as quickly as possible for job interview purposes, and as noted earlier, the presentations appear to have contributed to job opportunities for at least two postdoc participants.

### Final thoughts

This novel initiative is a tool for postdoctoral professional development that does not require an extended time commitment from postdocs, and it serves as a dynamic educational opportunity for the research community. It is a flexible model that can be easily adapted to the culture or needs of each institution, as VCU did after it learned of Pitt's program. Additionally, both programs have taken the opportunity to develop deeper institutional partnerships to strengthen their recruitment, promotion, postdoc support, and evaluation of the talk series. Health sciences libraries are encouraged to have discussions with their postdoctoral associations and Clinical and Translational Science Award (CTSA) programs to determine whether a similar series would benefit their research communities.

## Data Availability

Quantitative data associated with this article are available at scholarscompass.vcu.edu/libraries_data/4 in CSV format.

## SUPPORTING AGENCY

National Center for Advancing Translational Sciences

## SUPPLEMENTAL FILES

**Appendix A:** “How-to Talks by Postdocs”: survey

**Appendix B:** “How-to Talks by Postdocs”: speaker experience
