# Supplementary material for: Partnering with postdocs: a library model for supporting postdoctoral researchers and educating the academic research community
Source: J Med Libr Assoc. 2020 Jul 1;108(3):480–6. doi: 10.5195/jmla.2020.902 (PMC7441904; doi:10.5195/jmla.2020.902)
Supplement: Supplementary file 1 — Appendix A: “How-to Talks by Postdocs”: survey [file jmla-108-3-480-s01.pdf]

Journal of the Medical Library Association

Supplemental content to
J Med Libr Assoc. Jul;108(3):dx.doi.org/10.5195/jmla.2020.902
www.jmla.mlanet.org
© Gau, Dillon, Donaldson, Wahl, Iwema 2020

# Partnering with postdocs: a library model for supporting postdoctoral researchers and educating the academic research community

**Karen H. Gau; Pamela Dillon; Teraya Donaldson; Stacey E. Wahl; Carrie L. Iwema, AHIP**

## APPENDIX A

**"How-to Talks by Postdocs" survey**

Talk: ______________________________ Date: ______________________

Postdoc: ___________________________

Please rate your experience with this talk.

| | | | | | |
|---|---|---|---|---|---|
| I have a better understanding of this topic as a result of my attendance at this presentation. | Strongly disagree | Disagree | Neutral | Agree | Strongly agree |
| I plan to use the knowledge I learned. | Strongly disagree | Disagree | Neutral | Agree | Strongly agree |
| This was a good networking opportunity. | Strongly disagree | Disagree | Neutral | Agree | Strongly agree |

What did you enjoy about the talk?

_______________________________________________

Please evaluate the instructor.

| | | | | | |
|---|---|---|---|---|---|
| The instructor was well prepared. | Strongly disagree | Disagree | Neutral | Agree | Strongly agree |
| The instructor was responsive to questions. | Strongly disagree | Disagree | Neutral | Agree | Strongly agree |
| The instructor was an effective facilitator/teacher. | Strongly disagree | Disagree | Neutral | Agree | Strongly agree |

What were some of the instructor's strengths?

_______________________________________________

How could the instructor improve his/her teaching?

_______________________________________________

What other topics would you like to see included in the "How-to Talks by Postdocs" series?

_______________________________________________

How did you learn about this seminar?
☐ Email ☐ TelegRAM ☐ Colleague ☐ Website ☐ Other ______

What is your affiliation with Virginia Commonwealth University (VCU)?
☐ Undergraduate ☐ Graduate ☐ Postdoc ☐ Faculty ☐ Staff ☐ Other ______

Sponsored by VCU Libraries, Wright Center for Clinical and Translational Research, and the VCU Postdoctoral Association
