# Supplementary material for: Partnering with postdocs: a library model for supporting postdoctoral researchers and educating the academic research community
Source: J Med Libr Assoc. 2020 Jul 1;108(3):480–6. doi: 10.5195/jmla.2020.902 (PMC7441904; doi:10.5195/jmla.2020.902)
Supplement: Supplementary file 2 — Appendix B: “How-to Talks by Postdocs”: speaker experience [file jmla-108-3-480-s02.pdf]

JMLA Journal of the Medical Library Association

SC

Supplemental content to
J Med Libr Assoc. Jul;108(3):dx.doi.org/10.5195/jmla.2020.902
www.jmla.mlanet.org
© Gau, Dillon, Donaldson, Wahl, Iwema 2020

Partnering with postdocs: a library model for supporting postdoctoral researchers and educating the academic research community

Karen H. Gau; Pamela Dillon; Teraya Donaldson; Stacey E. Wahl; Carrie L. Iwema, AHIP

APPENDIX B

"How-to Talks by Postdocs": speaker experience

Thank you for participating in the "How-to Talks by Postdocs" series! Please fill out this evaluation to let us know about your experience in this program.

1. Which aspects of this series were helpful? You may select multiple answers.
- O Adding this talk to my resume
- O Getting feedback on my teaching skills
- O Watching a video recording of my talk (if applicable)
- O Participating in a dry run (if applicable)
- O Other (please specify)

2. Please rate how useful this series was in providing you with teaching experience.
O Useless O Not very useful O Neutral O Useful O Extremely useful

3. Please rate how useful this series was in providing you with a networking opportunity.
O Useless O Not very useful O Neutral O Useful O Extremely useful

4. Were the series coordinators responsive to your needs?
- O Yes
- O No
- O Other (please specify) ____________________

5. How could this series be improved?

____

6. Would you participate in this series again?
- O Yes
- O No
- O Other (please specify) ____________________

7. Would you recommend that other postdocs participate in this series?
- O Yes
- O No
- O Other (please specify) ____________________

8. Additional comments or suggestions?

____
